# Antagonistic Interactions Between *Dickeya solani* and *Bacillus subtilis*

**DOI:** 10.3390/ijms26157193

**Published:** 2025-07-25

**Authors:** Roberta Gatta, Adam Iwanicki, Robert Czajkowski, Michał Obuchowski

**Affiliations:** 1 Intercollegiate Faculty of Biotechnology UG-MUG, University of Gdańsk, 80-307 Gdańsk, Poland; roberta.gatta@phdstud.ug.edu.pl; 2 Institute of Medical Biotechnology and Experimental Oncology, Intercollegiate Faculty of Biotechnology UG-MUG, Medical University of Gdańsk, 80-210 Gdańsk, Poland; aiwanicki@gumed.edu.pl; 3 Laboratory of Biologically Active Compounds, Intercollegiate Faculty of Biotechnology UG-MUG, University of Gdańsk, 80-307 Gdańsk, Poland; robert.czajkowski@ug.edu.pl

**Keywords:** potato soft rot, phytopathogens, bacterial antagonism, LysR-family transcriptional regulator, swarming

## Abstract

Microorganisms in their natural ecological niches are constantly challenged by other inhabitants. Antagonisms exhibited by interacting microbial species are directed towards survival and increasing of their fitness. The Soft Rot Pectobacteriaceae (SRP) is a good model to study these complex microbial interactions. Along with being present in various environments, SRPs are often transferred between environments, allowing the bacteria to encounter members of other species. In this study, we investigated interactions between *Dickeya solani*, a representative of SRPs and a causative agent of potato soft rot, and *Bacillus subtilis*, which is known to be a potent producer of secondary metabolites mediating antibiosis. We have found that the soil isolate *B. subtilis* MB73/2 not only suppresses *in vitro* soft-rotting of infected potato tubers but is also able to cause directional, coordinated escape of natural isolates *D. solani* IFB0102 and IPO2222. While this coordinated movement of *D. solani* depends on surfactin produced by *B. subtilis* MB73/2, we show that both *Dickeya* strains exhibit different antagonistic interaction phenotypes toward the competing *Bacillus*. We prove that this antagonism depends on a single nucleotide polymorphism in one of transcriptional regulators of *D. solani* belonging to the LysR family.

## 1. Introduction

Microorganisms in their natural environment almost never exist alone. They rather function in multispecies communities in which they constantly face the need to interact with other inhabitants of the niche. Since they compete for nutrients and space, these interactions are multifaceted. The mechanisms underlying these phenomena, apart from trophic competition, are often based on production of toxic molecules or interference with signaling pathways [1].

A group of microorganisms referred to as the Soft Rot Pectobacteriaceae (SRP) [2] consists of pectinolytic Gram-negative bacteria responsible for causing diseases in a wide range of crops, ornamentals, and water environmental isolates. SRPs constitute a good model to study microbial interactions as the bacteria are present in various environments, such as soil, water, and host and non-host plants [3,4]. Moreover, they are often transferred from one environment to another, and therefore, they meet many other bacterial species and competitors. While many bacteria are capable of causing soft rot diseases in plants, the most-studied genera are *Pectobacterium* and *Dickeya*. They use similar virulence strategies, have overlapping hosts and geographical distributions, and are often found together in the environment [5]. Among twelve recognized species of *Dickeya* genus [6], *Dickeya solani* is given special attention by researchers. Within last two decades *D. solani* has become the predominant bacterial pathogen causing the potato blackleg in Europe, responsible for nearly 25% of incidences of this disease in the Netherlands, Belgium, and France [7].

*Bacillus subtilis* is a Gram-positive bacterium well known for its potential to produce secondary metabolites mediating antibiosis. Major classes of these metabolites include antimicrobial peptides (ribosomal and non-ribosomal), polyketides, and volatile compounds [8]. Apart from these, *B. subtilis* produces enzymes involved in the quenching of quorum sensing, which was shown to be especially important for interaction with SRPs, for example, *Pectobacterium carotovorum* subsp. *carotovorum*, a phytopathogen causing potato soft rot [9]. Members of the *Bacillus* genus are also capable of antagonistic interaction with different species of *Dickeya*. For example, *B. simplex* BA2H3 controls potato blackleg and soft rot diseases caused by *D. dianthicola* [10]; *B. cereus* BC3 antagonizes *D. zeae*, which causes bacterial heart rot in pineapple [11]; and culture supernatant of *B. amyloliquefacience* was shown to synergize with silver nanoparticles against *D. dadantii* [12].

In this work we studied the interaction of soil isolate *B. subtilis* MB73/2 with environmental strain *D. solani* IFB0102. *B. subtilis* MB72/3 was isolated from the meadow soil in Żuławy in Poland. It has been shown to be able to inhibit phytopathogens of genera *Dickeya* and *Pectobacterium* and to colonize the potato tuber rhizosphere following seed tuber bacterization [13]. *D. solani* IFB0102 was isolated in Poland from infected potato tubers. We show that *B. subtilis* MB72/3 suppresses *in vitro* soft-rotting of potatoes caused by *D. solani*. We demonstrate that the investigated *B. subtilis* strain exhibits a strong antagonistic effect against IFB0102 and triggers its directional movement on semi-solid media. Moreover, we prove that this movement is dependent on the presence of surfactin. At the same time, we show that the studied *Dickeya* strain is able to antagonize *B. subtilis* MB72/3, and we provide evidence that this phenomenon is strictly dependent on the LysR-family transcriptional regulator. While the nature of antagonistic interaction between *B. subtilis* and *D. solani* remains unknown, we provide evidence supporting the hypothesis that the observed phenomenon depends on both antagonizing species. Moreover, the genetic differences in *D. solani* strains underlie differential phenotypes.

## 2. Results

### 2.1. Bacillus Subtilis Suppresses In Vitro Soft-Rotting of Potatoes Caused by Dickeya solani

**Figure 1:**
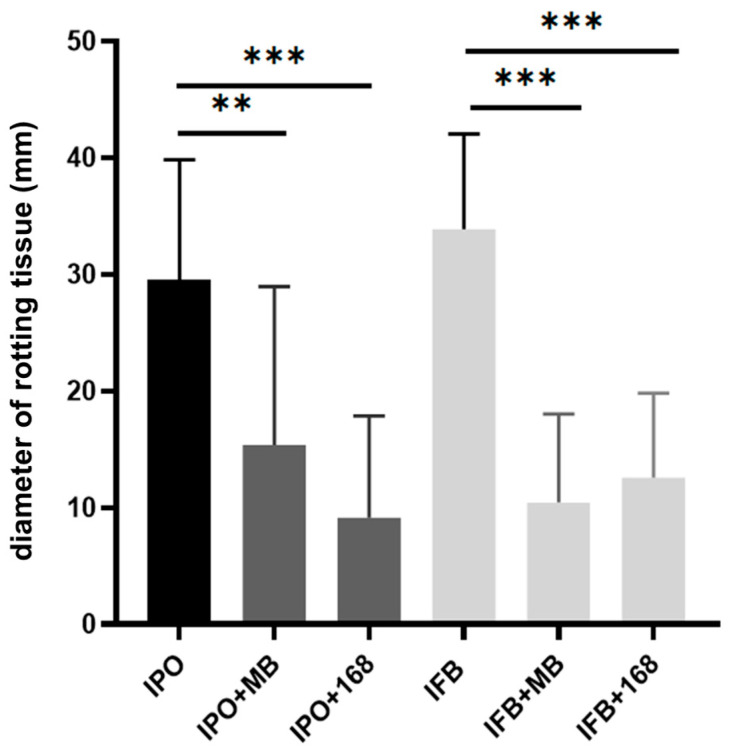
Diameter of rotting tissue (mm) in the potato slices infected with *Dickeya solani* IPO2222 (IPO) and IFB0102 (IFB) in comparison with the diameter of rotting tissue in the potato slices infected by *Dickeya solani* IPO2222 or IFB0102 in co-inoculum with *B. subtilis* MB73/2 (IPO+MB, IFB+MB) or 168 (IPO+168, IFB+168) 96h after infection. Error bars represent standard deviation. The experiment was repeated twice with three technical replicates. ** *p* < 0.01, *** *p* < 0.005.

*Bacillus subtilis* MB73/2 was shown to inhibit phytopathogens from genus *Dickeya* [14]. We wanted to check whether these bacteria are capable of protecting potato tubers from soft rot symptoms caused by *Dickeya solani*. The test was conducted using the method of co-inoculation of both strains on potato slices. The diameter of rotten tissue corresponded to the pathogenic effect of *D. solani*. We performed the test with *B. subtilis* MB73/2 soil isolate and the laboratory strain 168. Both strains exhibited protective effects against maceration of potato tuber tissue caused by *D. solani* IFB0102 and described the member of this species, *D. solani* IPO2222, as well [15] (Figure 1).

### 2.2. D. solani Strains IFB0102 and IPO2222 Exhibit Different Swarming Properties on Semi-Solid Medium

**Figure 2:**
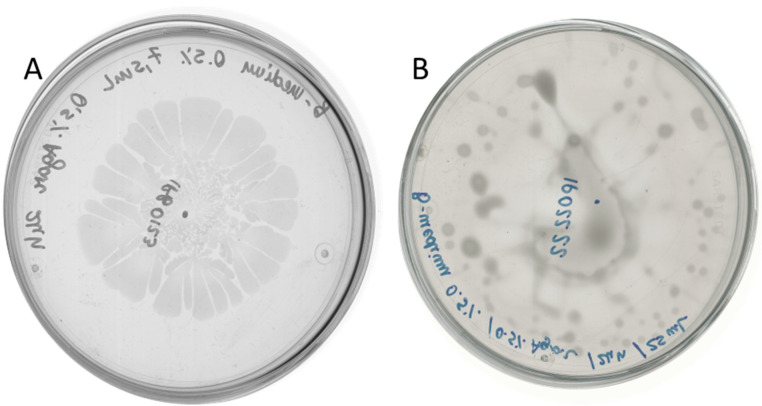
Swarming of *D. solani* IFB0102 (**A**) and IPO2222 (**B**) on 7.5 mL of 0.5x B-medium with 0.5% of agar 24 h upon inoculation.

To gain more insight into the interaction between *B. subtilis* and *D. solani* we wanted to analyze antagonistic effects caused by these bacteria co-cultivated on semi-solid medium. In our recent work we have performed in-depth investigation of *D. solani* swarming [16]. With the knowledge of the high variability of swarming outcomes, first we had verified the swarming capabilities of both tested *Dickeya* strains. *D. solani* IFB0102 exhibited a typical pattern with the formation of swarming dendrites departing from the inoculation point (Figure 2A), *D. solani* IPO2222 showed unidirectional irregular swarming and formation of a characteristic vortex pattern with dense clusters of cells on the agar surface, resulting in a notably moist environment within the plate (Figure 2B).

### 2.3. Bacillus subtilis and Dickeya solani Antagonize on Semi-Solid Medium

To investigate antagonistic interaction between *B. subtilis* and *D. solani* equal amounts (2 μL of refreshed culture at OD600 = 0.2) of *B. subtilis* MB73/2 and *D. solani* IFB0102 were inoculated in a Petri plate containing 0.5x concentrated B-medium supplemented with 0.5% of agar. Bacteria were inoculated at a distance of 1.5 cm, providing adequate space for the independent growth yet allowing interactions.

**Figure 3:**
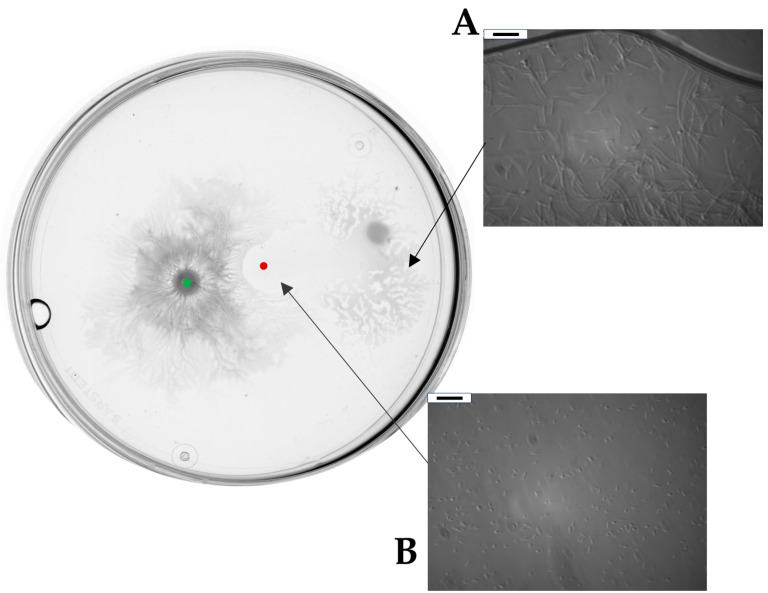
*B. subtilis* MB73/2 and *D. solani* IFB0102 social interaction on a swarming plate containing 0.5x B-medium with 0.5% of agar. Bacteria were inoculated at a distance of 1.5 cm and plate were observed 24 h after inoculation. Red dot indicates the inoculation point of *D. solani*. Green dot indicates point of inoculation of *B. subtilis*. (**A**) Example of magnification of inoculation zone under optical microscope. (**B**) Example of magnification of *D. solani* dendrites under phase contrast microscope. Scale bar: 10 μm.

After an incubation of 24 h, we could macroscopically register an architecturally complex phenotype. *B. subtilis* MB73/2 had created a highly wrinkled swarm pattern with a defined center and dense dendrites spreading from the central colony in all directions. Interestingly, the swarming of MB73/2 was interrupted at approximately 0.3 cm from the front of inoculation of *D. solani* IFB0102. Additionally, when the two colonies came into proximity, *D. solani* IFB0102 had swarmed in the opposite direction. Within a few hours, the central colony of *D. solani* IFB0102 was translocated entirely from the point of inoculation to the edge of the plate, moved by a distance of approximately 2.5 cm. Zooming in on the point of inoculation, we could have seen that the area of inoculation was inhabited by *D. solani* IFB0102 cells with a much lower density compared to the density of cells at the edge of the dendrites. In addition, cells at the inoculation point did not display the elongate swarm morphology (Figure 3).

**Figure 4:**
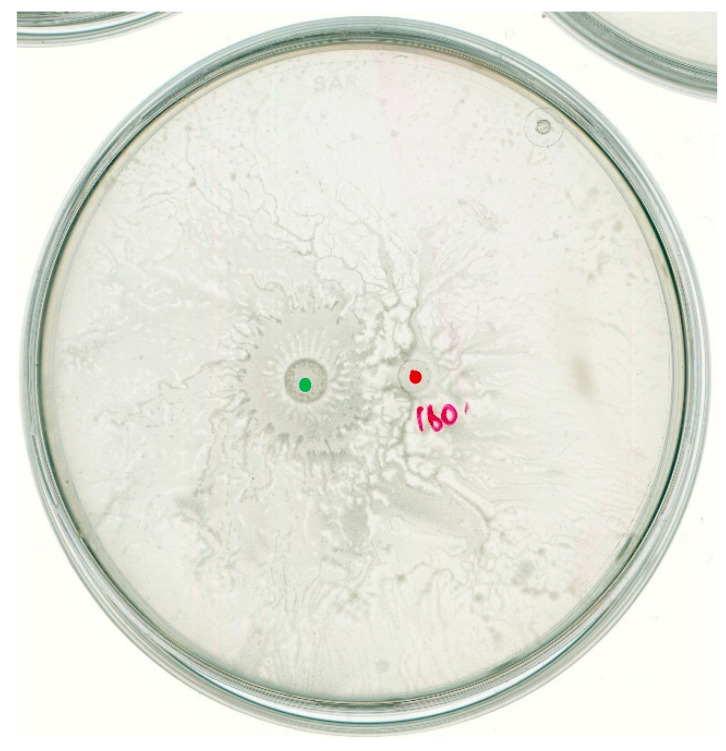
*B. subtilis* MB73/2 and *D. solani* IPO2222 social interaction on a swarming plate containing 0.5x B-medium with 0.5% of agar. Bacteria were inoculated at a distance of 1.5 cm. The observations were made 24 h after inoculation. Red dot indicates the inoculation point of *D. solani.* Green dot indicates the inoculation point of *B. subtilis*.

On the other hand, in the case of interacting *B. subtilis* MB73/2 and *D. solani* IPO2222 we could not observe any inhibition zone. The strain of IPO2222 exhibited directional movement away from the *B. subtilis* MB73/2. Nevertheless, the *B. subtilis* strain was able to colonize area previously occupied by *D. solani*, including its inoculation point (Figure 4).

### 2.4. Surfactin Is Required for Directional Escape of D. solani

**Figure 5:**
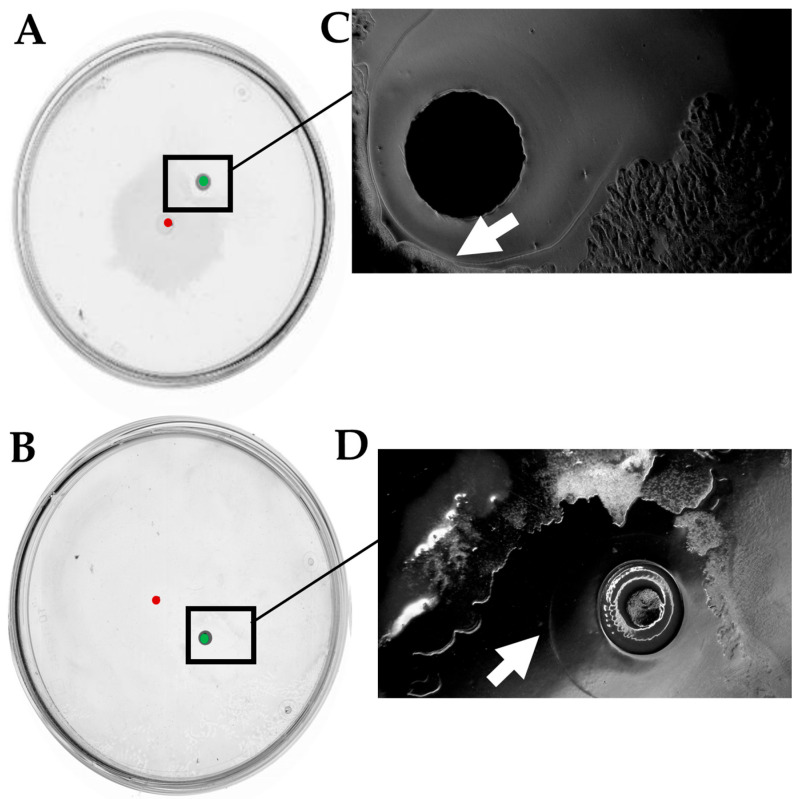
Swarming interaction of *D. solani* IFB0102 and (**A**) *B. subtilis* 168 or (**B**) *B. subtilis* MB73/2 *sfp*^−^ on 0.5x B-medium containing 0.5% of agar. (**C**) Interaction zone between *D. solani* IFB102 and *B. subtilis* 168. (**D**) Interaction zone between *D. solani* IFB0102 and *B. subtilis* MB73/2 *sfp*^−^. Red dots indicate points of inoculation of *D. solani*. Green dots indicate points of inoculation of *B. subtilis*. Arrows point to the definition of an inhibition ring.

The surfactin in some cases enable bacteria to swarm effectively on the solid surfaces. At the same time, it is known for its anti-microbial properties. Since *B. subtilis* is known to produce this compound, we wanted to investigate the importance for interaction between surfactant and the investigated strains of *B. subtilis* and *D. solani*. The production of surfactin in *B. subtilis* depends on the products of the *srfA* operon-*sfp* gene cluster. The Sfp protein (4-phosphopantetheinyl transferase) is essential for the surfactin synthesis [17]. However, in the laboratory strain *B. subtilis* 168 the *sfp* gene harbors an internal terminal codon, leading to the production of a truncated and nonfunctional Sfp protein. Therefore, the particulat one, *B. subtilis* 168 strain is not able to swarm under laboratory conditions at least on a reach medium. Taking it into the consideration, we have analyzed interaction of *D. solani* IFB0102 with *B. subtilis* 168. Upon the inoculation of *D. solani* strains on the same swarming plate with *B. subtilis* 168 we had observed independent growth of *Dickeya* in all directions. Nonetheless, once *Dickeya* encountered *B. subtilis* colony a prominent inhibition zone was formed around it (Figure 5A).

The closer examination of the point of interaction enabled us to observe that *B. subtilis* colony was surrounded by a ring which had exhibited altered light refraction. The ring delineated the confining zone that *D. solani* was unable to penetrate.

To extend our study, we created a mutant strain of *B. subtilis* MB73/2 by inactivating the *sfp* gene encoded in its genome. The mutant MB73/2 *sfp*^−^ showed no production of surfactin, confirmed by drop-collapsing assay [18], and did not swarm. The results of the interaction were very similar to those observed in the case of *B. subtilis* 168 (Figure 5B).

**Figure 6:**
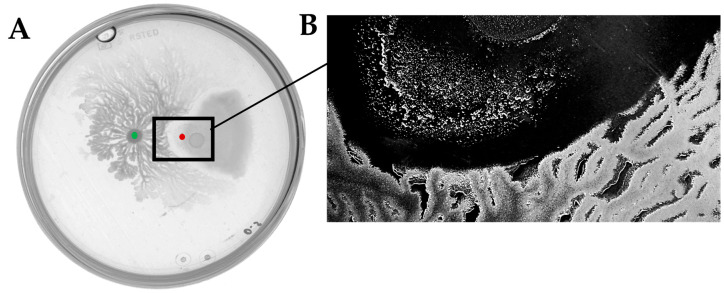
(**A**) Swarming interaction of *D. solani* IFB0102 and *B. subtilis* 168 *sfp*^+^ on 0.5x B-medium containing 0.5% of agar. (**B**) Inhibition zone appearing between the interacting bacteria and *D. solani* IFB0102 translocation from the point of inoculation. Red dot indicates the inoculation point of *D. solani.* Green dot indicates the inoculation point of *B. subtilis*.

To complete our experiments, we had used a modified strain *B. subtilis* 168 with reconstituted *sfp* gene (168 *sfp*^+^) [19]. The results of inoculation of either investigated *Dickeya* strain on the same plate with surfactin-producing strain *B. subtilis* 168 *sfp*^+^ resembled closely the results we had observed in the case of interaction with *B. subtilis* MB73/2. Again, the formation of an inhibition zone and the directional escape was observed (Figure 6).

**Figure 7:**
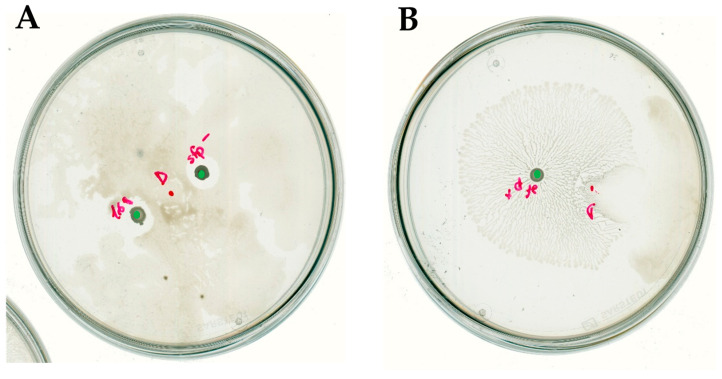
Swarming interaction of *D. solani* IPO2222 and (**A**) *B. subtilis* 168 and MB73/2 *sfp*^−^ or (**B**) *B. subtilis* 168 *sfp*^+^ on 0.5x B-medium containing 0.5% of agar. Red dot indicates the inoculation point of *D. solani*. Green dots indicate inoculation points of *B. subtilis*.

The interaction phenotype of *D. solani* IPO2222 was similar to *D. solani* IFB0102. In the case of co-inoculation with *B. subtilis* strains 168 and MB73/2 *sfp*^−^ we had observed independent growth of *Dickeya* along with a clear inhibition zone surrounding colony of *B. subtilis* (Figure 7A). When grown on the same plate with *B. subtilis* 168 *sfp*^+^, *D. solani* IPO2222 exhibited the directional escape with no visible inhibition zone (Figure 7B).

Overall, the above results suggest an important role of surfactin in the directional escape of *D. solani* in response to *B. subtilis.* Nevertheless, it is not responsible nor required for the formation of the inhibition zone between bacteria of the interacting species.

### 2.5. The sRNA ArcZ Is Not Responsible for Differences in Interaction of Investigated D. solani Strains with B. subtilis

A study by Brual and colleagues in 2023 provided insights into the interactions between *D. solani* strains and other microorganisms, including *B. subtilis*. In particular, they identified a single nucleotide polymorphism into the *arcZ* region (A at position 90 instead of G). ArcZ is a sRNA that works as a post-translational regulator. It was shown that this mutation at position 2530087 of *D. solani* IPO2222 genome is responsible for the loss of inhibition of *B. subtilis* [20]. These findings led us to investigate the sequence of *arcZ* in the strain *D. solani* IFB0102. We had performed whole genome sequencing both, IFB0102 and our version of the strain *D. solani* IPO2222. The consensus sequences were compared using the progressiveMauve algorithm. The sequences of both analyzed *D. solani* strains were identical with exception of 7 single nucleotide polymorphisms (Table 1).

**Figure 8:**
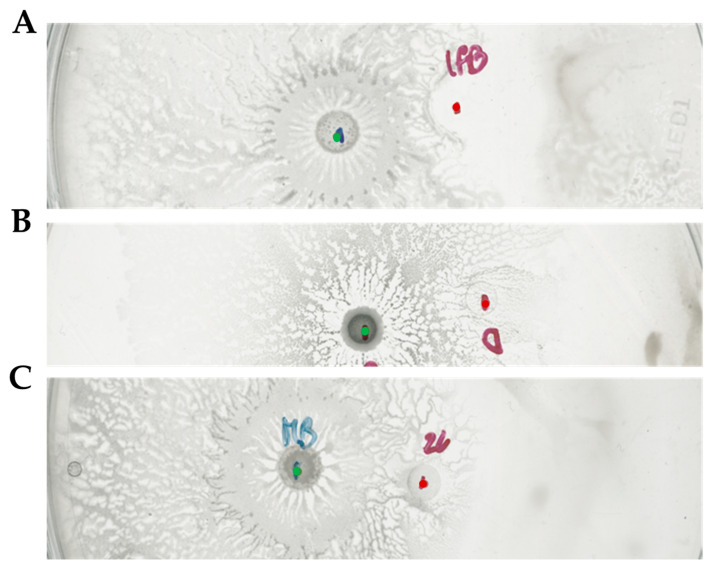
Swarming interaction between MB73/2 and IFB0102 (**A**), D s0432-1 (**B**), IPO2222 (**C**) on 0.5x B-medium containing 0.5% of agar. The inhibition zone is visible only in antagonism with IFB0102. Red dots indicate the point of inoculation of *D. solani* strains. Green dots indicate points of inoculation of *B. subtilis*.

Interestingly, one of these SNPs was in the mentioned above position 2530087 of the *D. solani* IPO2222 genome. Our version of IPO2222 harbored the same mutation reported in the *arcZ* region. At the same time, the sequence of *arcZ* present in the strain *D. solani* IFB0102 was identical to the sequence of the strain D s0432-1, which served as a wild type in the Brual’s work [20]. Considering the involvement of ArcZ in the interaction with *B. subtilis*, we had acquired strain *D. solani* D s0432-1 and proceeded to evaluate its ability to inhibit growth of *B. subtilis* MB73/2. Upon the interaction of these strains in a swarming assay, we were unable to detect any inhibition zone. Moreover, MB73/2 was able to colonize the area previously occupied by *D. solani* D s0432-1, which resembled the phenotype of *D. solani* IPO2222 used in our experiments (Figure 8).

The above-mentioned result led us to the conclusion that possibly other genes beyond *arcZ* are responsible for the impaired antagonism observed in the case the strain *D. solani* IPO2222.

### 2.6. The LysR Transcriptional Regulator Is Required for Antagonism Between D. solani and B. subtilis

The fact that investigated *D. solani* strains IFB0102 and IPO2222 exhibited differences in motility and antagonistic interaction with *B. subtilis* MB73/2 prompted us to consider the involvement of a transcriptional regulator in the regulatory network. One of the 7 SNPs differing genomic sequences of both analyzed *D. solani* strain is located at position 4635450, within the gene encoding a transcriptional regulator belonging to the LysR family. The LysR-type transcriptional regulators (LTTRs) currently represent the largest known family of bacterial regulators, comprising over 800 identified members [21]. The products of regulated genes serve various functions, encompassing cell metabolism, quorum sensing, virulence, motility and toxin production [22].

**Figure 9:**
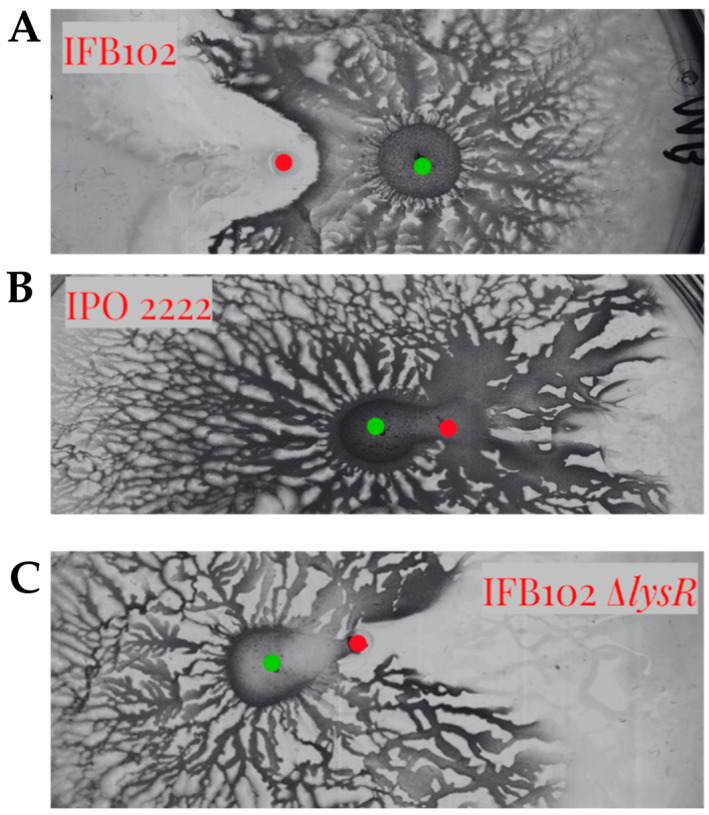
Swarming interaction between *B. subtilis* MB73/2 and *D. solani* IFB0102 (**A**), IPO2222 (**B**) and IFB0102Δ*lysR* (**C**) on 0.5x B-medium containing 0.5% of agar. The inhibition zone is not visible in both the mutant and the laboratory strain. Red dots indicate the point of inoculation of *D. solani* strains and green dots indicate the point of inoculation of *B. subtilis*.

To evaluate the role of the LysR-type regulator in the interaction between *D. solani* and *B. subtilis* MB73/2, we constructed a mutant strain of IFB0102 carrying a deletion of this gene. The deletion plasmid pUC19-Δ*lysR* plasmid was Gibson assembled in a way to enable the replacement of the *lysR* gene in the genome of *D. solani* with the gentamycin-resistance cassette. Upon the electroporation of *D. solani* IFB0102 we had obtained gentamycin-resistant colonies which carried deletion of the *lysR* gene. In the interaction with *B. subtilis* MB73/2, the *D. solani* IFB0102 Δ*lysR* strain have exhibited phenotype very similar to the *D. solani* IPO2222 phenotype, displaying enhanced motility and no inhibition zone (Figure 9).

The obtained results suggest strict requirement of the LysR regulator for antagonizing *B. subtilis* MB73/2 by interacting *D. solani*.

## 3. Discussion

The interaction between microorganism is an inevitable consequence of the complexity characterizing microbial communities existing in the nature. While some of these interactions may be beneficial to each member of the community, the antagonistic interactions are common. It is a consequence of competition for scarce available resources in the given ecological niche. The strategies utilized by antagonizing microorganisms include production of metabolites and products toxic to other microbes, trophic competition or signaling-based interactions [1].

The research of microbial interactions may provide valuable information, which can be exploited to combat the pathogenic species. Such approach is especially interesting in the context of biocontrol of plant pathogens, which can have a long-term significant impact on economy and nutritional safety of human population. With the beginning of the XXI^st^ century, the significance of *Dickeya solani* as potato blackleg pathogen started to grow [7]. In light of the unsatisfying efficiency of traditional plant protection methods, the biocontrol approach seems to be an interesting alternative in combating infections with this phytopathogen [23]. At present, *Bacillus* species are by far the most widely used bacteria in bioformulations due to their ability to form endospores that can resist biotic and abiotc stress, secrete a wide range of antimicrobial compounds and enhance plant growth and soil health [24].

*D. solani* strains exhibit an exceptionally high level of genome homogeneity, which is underscored by ANIb values ranging from 98.55% to 99.93% and ANIm values ranging from 98.71% to 99.92% [25]. Despite this high level of genomic identity, *D. solani* strains were found to vary significantly in virulence, production of plant cell wall-degrading enzymes and motility [26]. Taking it into account, the observed differences in phenotypes exhibited by *D. solani* strains IFB0102 and IPO2222 investigated in the presented study, are not surprising. Especially, considering the nearly complete identity of genomic sequences. The following processes like swarming motility or interaction with other bacteria, depend on multiple factors and genes. Therefore, it was expected that such significantly different properties of these strains, along with detected only 7 SNPs differing their genomes, should depend on changes in sequence of genes encoding some pleiotropic transcriptional regulators. Indeed, products of two genes encoded in genomes of *D. solani* IFB0102 and *D. solani* IPO2222, in which we have detected SNPs, are involved in regulation of various physiological processes of the studied bacteria. The polymorphism in the sequence of *arcZ* gene encoding small regulatory RNA reported to be responsible for the loss of inhibition of *B. subtilis* [20] turned out to have no impact on observed antagonism between both analyzed species. While it may seem to contradict the original finding by Brual et al. [20] it can be explained in two ways. Firstly, the *B. subtilis* strain used in Brual’s work was the laboratory strain PY79, whereas we used the environmental strain MB73/2. Hence, it seems plausible that these two strains exhibit different responses to antimicrobials produced by *D. solani*. Secondly, the inhibition of growth in Brual’s work was verified with different method, i.e., using a spot-on-lawn assay. In this assay bacteria do not establish any form of motility. In our experiments we had analyzed interactions of investigated strains in swarming assays in which bacteria are in the state of active movement. Another *D. solani* gene in which we detected a sequence polymorphism encodes LysR-familiy transcriptional regulator. Members of this family were shown to be important for virulence of these bacteria [27]. Therefore, our observation that the lack of antagonism exhibited by *D. solani* IPO2222 towards *B. subtilis* MB73/2, which was noticed for *D. solani* IFB0102, can find its explanation in the alteration of amino acid sequence of this LysR-family protein.

Interestingly, another gene in which we have detected a polymorphism in *D. solani* IFB0102 and *D. solani* IPO2222 was a HNH/endonuclease VII fold putative polymorphic toxin. The proteins that belong to this family are involved in contact-dependent growth inhibition (CDI), originally discovered in *Escherichia coli* strain EC93. In this strain CDI is mediated by the CdiA/CdiB two-partner secretion system, where CdiB is required for assembly of the CdiA onto the outer membrane. In EC93 CDI-mediated growth inhibition coincides with disruption of the proton motive force across the cell membrane, decreased aerobic respiration, and decreased ATP levels in the target cells [28]. Cells are protected from autoinhibition by an immunity protein CdiI [29], which either binds CdiA or neutralizes the growth inhibitory signal [30]. In *Dickeya dadantii* EC16, lack of a putative CdiI protein, designated VirA, reduces virulence on plant hosts [31]. Other results suggest that this protein may play a vital role in intra-species growth competition in the environment [30]. Taken together, our observations suggest an important role of proteins that belong to the discussed family in physiology of bacteria’s interaction. While such hypothesis is intriguing, the protein seems to actually be unrelated to the observed differences in antagonism of investigated *D. solani* strains against *B. subtilis* MB73/2. Although the sequence of the HNH/endonuclease VII fold putative polymorphic toxin in the strain *D. solani* D s0432-1 is identical to that encoded in the genome of *D. solani* IFB0102. Also, D s0432-1 exhibited the same phenotype of interaction with *B. subtilis* MB73/2 as the strain *D. solani* IPO2222.

Swarming motility of bacteria necessate presence of the surfactant [32]. Many swarming bacteria synthesize and secrete surfactants which, by reducing tension between bacteria and the surface, enable their spread. While the various isolates of *D. solani* were shown to produce no surfactants, some mutants in *pecS* or *pecT* genes were reported to do so [33]. PecS protein is a global regulator of the symptomatic phase of the disease caused by infection of plants with *D. dadantii* 3937. It controls the production and secretion of plant cell wall-degrading enzymes (PCWDE), as well as pigment, flagella and biosurfactants [34,35]. PecT is another main virulence negative regulator which controls synthesis of PCWDE and exopolysaccharide. It was also shown to function as the thermoregulator of the target genes [27].

Since *B. subtilis* is known to be a potent producer of surfactin, the interacting *D. solani* strains may make use of the produced surfactant in the plates co-inoculated with these bacteria. In our experiments surfactin did not seem to act as a repellent of *D. solani*. Rather, it has permitted the interacting bacteria to swarm all over the agar. Moreover, the presence of surfactin produced by *B. subtilis* MB73/2 might increase permeability of *D. solani* membrane to AHL molecules thereby fostering a faster and well-coordinated escaping of these bacteria.

The nature of antagonistic interaction between investigated strains of *B. subtilis* and *D. solani* remains unknown. Well known properties of *B. subtilis* strain like production of various antimicrobial compounds leaves room for speculation on the role of individual agents responsible for the directional escape of *D. solani*. The identification of the actual *B. subtilis* product/products associated with the observed phenomenon would require more systematic analysis, which is beyond scope of this study. Also understanding the differences in phenotypes of *D. solani* IFB0102 and IPO2222 requires further research. While the presence of the functional LysR-family transcriptional regulator was unambiguously shown to be crucial for antagonism exhibited by *D. solani* towards *B. subtilis*, the exact mechanism of this phenomenon is unknown. It seems plausible that the change in amino acid sequence of this protein, which was detected in both analyzed *D. solani* strains, may be responsible for alteration of the exhibited phenotype. The LysR-family regulators, besides involvement in other physiological processes, are responsible for regulation of motility and biofilm formation in *Agrobacterium tumefaciens* [36,37,38] or *Yersinia pseudotuberculosis* [39]. In *Escherichia coli*, proteins from this family were shown to regulate biosynthesis of flagella [40]. An interesting model of signaling circuit involving ArcZ sRNA and PecT transcriptional regulator was proposed by Yuan et al. [41] for another species of *Dickeya*, *D. dadantii*. According to this model, ArcZ negatively regulates *pecT* expression by targeting mRNA of this gene. The action of ArcZ requires presence of the chaperone Hfq [42]. The PecT represses transcription of *rsmB* gene encoding another sRNA responsible for regulation of genes which products are involved in formation of type III secretion system of *D. dadantii* important for full virulence of this bacterium [43,44]. Moreover, Hfq-mediated regulation of RsmB expression is dependent on c-di-GMP signaling pathways. The c-di-GMP signaling is also crucial of Hfq-dependent regulation of swimming motility of *D. dadantii*. Hence, a combined action of ArcZ and LysR-family regulator PecT can regulate both the virulence and motility of this bacterium. While our results suggest no direct contribution of ArcZ to the observed phenotype of *D. solani*, the mechanism described above supports our findings made on the role of LysR-family regulator in shaping of the interaction between both analyzed bacterial species.

A transcriptomic analysis of investigated *D. solani* strains, by providing deeper insight into the gene expression patterns, may help to understand the differences in observed phenotypes and contribute to explanation of antagonism that these bacteria exhibit towards *B. subtilis*.

## 4. Materials and Methods

### 4.1. Bacterial Strains and Media

Strains used in this study are listed in Table 2.

All strains were cultured in Luria broth (LB) medium (tryptose 10 g/L, yeast extract 5 g/L, NaCl 10 g/L) supplemented with antibiotic when required. The temperature of growth was set at 28 °C for *D*. *solani* and at 37 °C in the case of *B*. *subtilis* and *E. coli*. Swarming motility was performed on synthetic B-medium [52] which contains 15 mM (NH_4_)_2_SO_4_, 8 mM MgSO_4_, 27 mM KCl, 7 mM sodium citrate, 50 mM Tris/HCl (pH 7.5) supplemented on the day of inoculation with 0.6 mM KH_2_PO_4_ 2 mM CaCl_2_, 1 μM FeSO_4_, 10 μM MnSO_4_, 4.5 mM glutamic acid, 0.78 mM tryptophan, 0.8 mM Lysine and 0.5% (*w*/*v*) glucose. For the routine cultivation of bacteria, the medium was solidified with 1.5% (*w*/*v*) of Bacto agar. The swarming plates were prepared by supplementing the medium with 0.5% (*w*/*v*) of Bacto agar.

### 4.2. Screening for Antagonistic Interaction on Potato Slices

The screening for the ability to attenuate potato tissue maceration by *B. subtilis* strains was conducted following the method outlined by Jafra et al. [53], with necessary modifications. Initially, potato tubers were surface sterilized using 5% sodium hypochlorite for 10 min, followed by rinsing twice with sterile water. After air-drying for 2 h, the tubers were sliced into 1.5 cm thick slices. Using a sterile cork borer, three wells measuring 9 mm in diameter and 10 mm in depth were made in each slice. *B. subtilis* and *D. solani* strains were cultured overnight and refreshed in new LB medium the following morning. Subsequently, the wells were filled with 50 μL of a mixture containing equal parts (1:1 ratio) of *B. subtilis* and *D. solani* strains at OD600 of 0.1. The control potato slices were inoculated with either water or mono-cultures of *B. subtilis* and *D. solani*. The potato tuber slices were then placed in sterile 25 cm glass plates filled with 10 mL of water to create a moist environment. The plates were incubated at 28 °C for 72 h. The diameter of rotting tissue was measured. The statistical analysis of obtained data was performed using GraphPad Prism ver. 9 (GraphPad Software, Boston, MA, USA). The Shapiro-Wilk test was utilized to assess the normal distribution of the data. The homogeneity of variance was analyzed using the Fisher-Snedecor test. One-way ANOVA followed by Tukey’s post hoc tests was applied to evaluate differences between analyzed samples.

### 4.3. Swarming Motility

A single colony of investigated bacterial strain was inoculated in LB medium and incubated overnight with shaking (160 rpm) at 28 °C for *D. solani* and at 37 °C for *B. subtilis*. Two microliters of the overnight culture (OD600 ≈ 0.8, approx. 8 × 10^8^ CFU/mL) were inoculated in the center of a plate containing 7.5 mL of B-medium (0.5% of agar) and incubated for 24 h or 48 h at 28 °C (relative humidity 80% saturation). Plates were prepared 1 h before the inoculation and dried open for 30 min in a laminar flow chamber.

### 4.4. Swarming Motility Screening of Antagonistic Interaction

Single colonies of *D. solani* and *B. subtilis* were transferred in separate LB flasks and cultivated overnight at 28 °C and 37 °C, respectively. On the day of the experiment, the plates containing 7.5 mL of 0.5x B-medium with 0.5% of agar were prepared one hour prior to inoculation. The plates were dried for 30 min. *B. subtilis* and *D. solani* were inoculated on the same swarming plate at a distance of 1.5 cm. The plates were incubated at 28° C with the lids facing downward (relative humidity at least 80% saturation). The day after, the swarming interaction pattern was visualized with high resolution camera, Optilia W30x-HD, documenting each plate. The plates were scanned to store images. Each experiment was repeated three times, using mono-species swarming plates as controls.

### 4.5. A Drop-Collapsing Test for Biosurfactant Production

The production of biosurfactants was analyzed using modified drop-collapsing test [18]. 1 mL of overnight culture broth was centrifuged at 5000× *g* for 10 min at 4 °C and the supernatant was collected for the test. 10 mL of sterile water was poured into a Petri plate and 20 μL of mineral oil was carefully added to the water surface. Next, 5 μL of culture supernatant was spotted into the oil. Incase off biosurfactants presence, the oil drop would disrupte rapidly within seconds.

### 4.6. Construction of Plasmids

For inactivation of *B. subtilis* MB73/2 *sfp* gene its internal fragment of 520-bp was PCR amplified using sfp-F and sfp-R primers and MB73/2 genomic DNA as a template (Table 3).

The PCR product was sequentially digested with BamHI and SalI restriction enzymes cloned into the pMutin4 vector yielding pMutin-*sfp* plasmid. For deletion of *D. solani* IFB0102 *lysR* Gibson assembly technique was used. Two 500 bp fragments flanking *lysR* gene were PCR amplified using primers LysRleft-F, LysRleft-R, LysRright-F and LysRright-R and *D. solani* IFB0102 genomic DNA as a template. The gentamycin resistance cassette was PCR amplified using Gent-F and Gent-R primers and pKNOCK-Gm plasmid DNA as a template. Obtained fragments were assembled with pUC19 plasmid digested with KpnI and BamHI using Gibson Assembly Master Mix (New England Biolabs, Ipswich, MA, USA) following manufacturer’s protocol yielding pUC-Δ*lysR* plasmid. As a host for cloning, *Escherichia coli* strain DH5α (Table 2) was used. Bacterial strains were transformed using CaCl_2_-mediated transformation of *E. coli* as previously described [54].

### 4.7. B. subtilis Transformation

A single colony of *B. subtilis* MB73/2 was inoculated in minimal salts medium (MSM) (0.2% (NH_4_)_2_SO_4_, 1.4% K_2_HPO_4_, 0.6% KH_2_PO_4_, 0.1% sodium citrate, 0.02% MgSO_4_, 0.5% glucose, 4% tryptophane, 0.02% casamino acids, 2 mg of ferric ammonium citrate per liter) without antibiotic and incubated overnight at 37 °C with shaking. The day after, the culture was diluted 1:10 in fresh MSM medium and incubated at 37 °C with shaking for 3 h. Then, the culture was diluted 1:1 with starvation medium (0.2% (NH_4_)_2_SO_4_, 1.4% K_2_HPO_4_, 0.6% KH_2_PO_4_, 0.1% sodium citrate, 0.02% MgSO_4_, 0.5% glucose) and further incubated for 2 h at 37 °C with shaking. Following the starvation period, 1 μg of DNA was added to 100 μL of the cell suspension, and the mixture was incubated at 37 °C with shaking for 30 min to facilitate DNA uptake. To induce phenotypic resistance expression, the suspension was then diluted 1:4 in LB medium and incubated for 45 min at 37 °C with shaking. At the end of the incubation, cell suspension was plated on previously prepared LB agar plates supplemented with proper antibiotic for phenotypic selection. Plates were incubated overnight at 37 °C.

### 4.8. Preparation of D. salami Competent Cells

A single colony of *D. solani* was spread on a tryptic soy agar (TSA, Oxoid) plate and incubated overnight at 28 °C. After 48 h, all bacterial colonies were scraped from the plate and resuspended in 1 mL of 10% sterile glycerol solution. The cell suspension was washed in 10% glycerol (8000× *g* 5 min at 4 °C). After each washing step, cells were resuspended in a lower volume (1 mL, 0.5 mL, 0.25 mL and 20–30 μL). Cells were stored on ice and use immediately for electroporation.

### 4.9. D. solani Electroporation

Competent cells (20–30 μL aliquots) were mixed with 1 μg of purified plasmid DNA in cooled 0.1 cm Bio-Rad Gene Pulser electroporation cuvettes (Bio-Rad, Herculies, CA, USA) and incubated on ice for 1 h. The cell-DNA mixture was then electroporated using a MicroPulser Electroporator (Bio-Rad, Herculies, CA, USA) at 2.5 kV for 1–2 s. Immediately after electroporation, 500 μL of cold LB medium was added for cell recovery, followed by incubation at 28 °C for 1–2 h. Transformed cells (100 μL) were plated onto LB agar plates with antibiotic and incubated for 48 h at 28 °C.

### 4.10. D. solani IFB0102 Genome Sequencing and Analysis

Genomic DNA of *D. solani* IFB0102 was purified using a E.Z.N.A. Bacterial DNA Kit (Omega Bio-tek, Norcross, GA, USA) following the manufacturer’s instructions. Whole-genome sequencing was performed by Genomed S.A. (Warsaw, Poland) on an Illumina MiSeq platform. The high-quality paired-end reads were assembled de novo using SPAdes v. 3.14.1 and IPO2222 genome sequence (GenBank accession no CP015137) as reference. The resulting consensus sequence was automatically annotated in the process of deposition in the GenBank database under accession number CP183043. The pairwise alignment of genomic sequences of strains IFB0102 and IPO2222 generated using progressiveMauve algorithm (Mauve v. 2.4.0) [55] was used for identification of SNPs.

## 5. Conclusions

In conclusion, our data show the ability of the environmental strain *B. subtilis* 73/2 to limit the growth of *D. solani* IFB0102. The combined results highlight the complexity of regulatory mechanisms underlying antagonisms between investigated bacteria. Observed differential interaction and motility of studied strains of *D. solani* emphasize the importance of differences found in their genomes. The capability of *B. subtilis* MB73/2 to suppress soft-rotting of potato tubers caused by *D. solani* provides good prospects for use of this strain in agricultural applications.

## Figures and Tables

**Table 1:** List of the SNPs between *D. solani* IPO2222 and *D. solani* IFB0102. The table reports the position in the IPO2222 genome, the type of substitution and the product of the gene.

SNPs	Position	IPO2222	IFB102	Product
1	2530087	T	C	ArcZ
2	2621920	C	T	Hypothetical protein kinase
3	3554462	C	A	cytochrome d terminal oxidase subunit 1
4	3674549	A	G	HNH/endonuclease VII fold putative polymorphic toxin
5	4039850	G	A	intergenic region
6	4513052	A	G	Hypothetical protein
7	4635450	C	T	transcriptional regulator-LysR family

**Table 2:** Bacterial strains and plasmids used in this study.

Strain	Relevant Characteristics	Reference or Source
*E. coli*		
DH5α	*F-gyrA96 recA1 relA1 endA1 thi-1 hsdR17 glnV44 deoR D (lacZYA-argF) U169[f80dD(lacZ)M15]*	[45]
*B. subtilis*		
MB73/2	Natural isolate	[46]
MB73/2 *sfp*^−^	*sfp::pMutin4*	This work
168	*trpC2*	[47]
168 *sfp*^+^	*trpC2 amyE::sfp*	[19]
*D. solani*		
IFB0102	Natural isolate	[48]
IFB0102 *lysR*	*lysR::pMutin4*	This work
IPO2222	Natural isolate	[15]
D s0432-1	Natural isolate	[20]
Plasmid		
pMutin4	Integration vector used for gene inactivation; Amp^r^ Ery^r^	[49]
pMutin-*sfp*	pMutin4 derivative carrying a PCR product internal to *sfp*	This work
pUC19	Cloning vector; Amp^r^	[50]
pKNOCK-Gm	Suicide vector for gene knockout; Gm^r^	[51]
pUC-Δ*lysR*	pUC derivative carrying a gentamycin resistance cassette flanked by PCR products of upstream and downstream regions of *lysR* gene	This work

**Table 3:** Primers used in the study.

Amplified Gene	Name	Sequence
*sfp*	sfp-F sfp-R	attaGGATCCACGGTTCATGTCTTTCATATC attaGTCGACGATATAGCATGGGGAATGG
*gentamycin*	Gent-F Gent-R	gagaggattcgagAGGACGCGTCAATTCTCG gtattacaaggctTAACAGATGAGGGCAAGC
*LysR-Left*	LysRleft-F LysReft-R	cggccagtgaattcgagctcggtacAAGCTTGTTTCGGTGTTGttgacgcgtcctCTCGAATCCTCTCGTATTATTTTC
*LysR-Right*	LysRright-F LysRright-R	cctcatctgttaAGCCTTGTAATACGGTCC gcatgcctgcaggtcgactctagagCTATTCTAATTCGTTCCGTTG

All primers were designed in the study.

## Data Availability

The authors confirm that the data supporting the findings of this study are available within the article.
